# Re-calibrating measurements of low-cost air quality monitors using PCR-GPR air quality forecasting models

**DOI:** 10.1371/journal.pone.0314417

**Published:** 2025-02-06

**Authors:** Bing Liu, Shuting Yang, Junqi Wang

**Affiliations:** 1 Public Foundational Courses Department, Nanjing Vocational University of Industry Technology, Nanjing, China; 2 Research and development department, Nanjing Changyang Technology Development Company Limited, Nanjing, China; 3 School of Electrical Engineering, Nanjing Vocational University of Industry Technology, Nanjing, China; University of Memphis, UNITED STATES OF AMERICA

## Abstract

As a key tool for real-time monitoring of air pollutant concentrations, the chemical sensor, the core component of the low-cost Air Quality Monitor (AQM), is susceptible to a variety of factors during the measurement process, leading to errors in the measurement data. To enhance the measurement accuracy of chemical sensors, this paper presents a calibration method based on the PCR-GPR model. This method not only effectively enhances the measurement accuracy of chemical sensors, but also combines the interpretability of traditional statistical models with the high-precision characteristics of Gaussian Process Regression (GPR) models. First, we perform Principal Component Analysis (PCA) on the measurement data of the AQM to solve the multicollinearity problem. Through PCA, we successfully extracted 8 principal components, which not only contained 95% of the information in the original data, but also effectively eliminated the correlation between the variables, providing a more robust data base for subsequent modeling. Subsequently, we established a Principal Component Regression (PCR) model using the concentration of pollutants measured by the national monitoring station as the dependent variable and the 8 principal components extracted above as the independent variables. The PCR model can effectively extract the linear relationship between the independent and dependent variables, providing a linear part of the explanation for the calibration process. However, there are often complex nonlinear relationships between pollutant concentrations and AQM measurements. To capture these nonlinear relationships, we further established a GPR model with the residuals of the PCR model as the dependent variable and the measurement data of the AQM as the independent variable. By combining the PCR model and the GPR model, we obtained the final PCR-GPR calibration model. It is worth mentioning that this study adopted the time series cross-validation method for data grouping, an innovative approach that is more aligned with real-world scenarios and adequately captures the seasonal variations in pollutant concentrations. The experimental results show that the model exhibits excellent performance on several evaluation metrics and can calibrate the chemical sensor well, improving its measurement accuracy by 16.94% ~ 82.01%.

## 1. Introduction

### 1.1. Chemical sensors for air quality monitoring

With the rapid development of industrialization and urbanization, air quality problems have become more and more prominent, causing serious impacts on public health and environmental quality [1, 2]. Therefore, air quality monitoring has become one of the important tasks in environmental protection and urban management. As a portable and real-time air quality monitoring tool, the low-cost Air Quality Monitor (AQM) has received wide attention and application. Chemical sensors (including electrochemical sensors, particulate sensors, etc.), as the core components of AQMs, have the advantages of fast response speed, high sensitivity and low cost, and play an important role in air quality monitoring [3].

However, a number of challenges remain for chemical sensors in practical applications. First, the measurement data from chemical sensors are often subject to errors and uncertainties due to the complexity of environmental factors and the measurement limitations of the chemical sensors themselves. This leads to the limited measurement accuracy of AQMs, which makes it difficult to meet the demand for high-precision monitoring [4]. Secondly, the measurement differences between different sensors also increase the complexity of data processing. Therefore, calibrating and correcting the measurement data of chemical sensors to improve the measurement accuracy of AQMs has become one of the focuses of current research.

In recent years, Radio Frequency Identification (RFID) technology has demonstrated extensive application potential across various sensors, particularly in the area of structural health monitoring. Studies have shown that RFID strain sensors, which monitor and transmit strain data wirelessly, significantly reduce the wiring complexity and maintenance costs associated with traditional wired sensors. Relevant research indicates that RFID strain sensors employing a dual-interrogation mode offer advantages such as low power consumption, high transmission distance, and temperature self-compensation, making RFID strain sensors effective in dynamic and complex environments [5, 6]. Although RFID technology has shown advantages in certain domains, the advancement of data processing techniques for chemical sensor calibration in air monitoring remains a crucial research focus today. Additionally, a study evaluating five low-cost air quality sensors found that the HPMA115 sensor performs excellently in both indoor and outdoor environments, making it suitable for air quality monitoring, but further sensor calibration is needed to enhance its reliability [7].

Existing air quality monitoring networks rely mainly on National Monitoring Stations (NMSs), which are accurate in their measurements but costly and complicated to maintain, making it difficult to realize large-scale grid arrangements. At the same time, the lag in data dissemination limits their ability to provide immediate feedback and monitor air quality. Therefore, more effective monitoring methods and technologies need to be explored to enhance the timeliness and broad spectrum of environmental monitoring [8, 9].

The wide application of AQMs provides a new solution for air quality monitoring. Its working principle is based on a variety of sensor technologies, including electrochemical sensors, particulate sensors and physical sensors. Electrochemical sensors use electrochemical reactions to convert the concentration of ambient gaseous pollutants into a data output, particulate sensors are specifically designed to measure the concentration of particulate matter, and physical sensors are used to monitor meteorological parameters. AQMs use these sensors in conjunction with each other to achieve real-time monitoring of major pollutants and meteorological parameters [10]. The deployment of multiple AQMs in a key area enables grid monitoring of the area. Typically, AQMs are calibrated to factory standards prior to deployment. For example, PM_2.5_ and PM_10_ are typically initially calibrated using standard particulate matter of known mass concentration, while CO, NO_2_, SO_2_, and O_3_ are typically calibrated using standard gases of known concentration. However, the measurements of AQMs are affected by weather factors, unconventional pollutants, and the drift of the chemical sensors themselves, which makes their measurement error problem still prominent [11].

### 1.2. Air quality forecasting models

Air quality forecasting models are an effective tool for predicting and simulating future air quality by combining a large amount of monitoring data and meteorological information. When calibrating the measurements of AQMs, these models can provide effective reference data to help analyze and adjust its measurements. By comparing with the actual monitoring data, the forecasting models can identify possible errors or deviations of the AQMs, and then adjust or calibrate the output of the sensors to improve the accuracy and reliability of the measurements. Among them, mechanistic and statistical models are common forecasting models. Mechanistic models simulate the physicochemical processes of pollutants by combining meteorological principles with mathematical methods, and use chemical equations to describe the generation and disappearance of pollutants, taking into account factors such as chemical reactions, turbulent diffusion, and radiation, to predict the air quality and the concentration of pollutants in the atmosphere [12, 13]. The mechanism model has a certain meteorological and chemical theoretical basis, which can deeply understand the concentration of pollutants in the atmosphere, but due to the complexity of the process of pollutant formation and propagation, there is room for improvement of the accuracy of the mechanism model.

Statistical modeling also has a wide range of applications in air quality prediction. Most of the traditional statistical models are based on historical observation data and statistical methods to predict air quality by analyzing the statistical relationship between the concentration of air pollutants and their influencing factors. The more common statistical models include techniques such as regression analysis [14, 15], Gray Prediction [16], Hidden Markov Chain [17, 18], and time series analysis [19]. Based on the historical data of Shanghai from March 2016 to February 2018, Gu et al. established a fuzzy multiple linear regression model and successfully realized the prediction of air quality index [20].

In recent years, machine learning and neural network techniques have been widely explored in the field of air quality prediction. Neural networks are able to learn and understand the complex relationships between different pollution factors through multi-level data processing and pattern recognition. A trained neural network model can predict air quality for a future period of time based on factors such as geographic location, environment, time of day, and meteorological parameters [21–23]. In addition, machine learning algorithms such as Random Forest [24–26], Gaussian Process Regression (GPR) [27] and Support Vector Regression (SVR) [28, 29] are widely used for air quality prediction. These algorithms analyze historical data and build models to predict future air quality conditions. Sachdeva et al. proposed a comprehensive framework for predicting the air quality index using pollutant concentration data and meteorological data, and the results of the study showed that different methods were applicable to different pollutants, among which the ARIMA model and artificial neural network were more effective in prediction [30]. Suriano et al. calibrated CO and NO_2_ concentrations measured indoors by AQMs using machine learning and neural network techniques, and the results of the study showed good agreement between the measurements of the calibrated AQMs and the data from a reference instrument [31]. Borah et al. combined machine learning (ML) and deep learning (DL) techniques to construct a hybrid ensemble model for air quality prediction in Kuala Lumpur, and the results of the study demonstrated that the model achieved high accuracy in predicting the concentrations of six major air pollutants, with R^2^ scores ranging from 0.87 to 0.97 [32]. Patra et al. developed an artificial neural network model to correlate PM concentrations with meteorological parameters, and the results showed strong agreement between the experimental data and the modeled output [33].

GPR is frequently employed in air quality forecasting due to its ability to handle nonlinear data and provide uncertainty estimates. Liu et al. proposed a soft sensor utilizing GPR that combines the squared exponential covariance function and periodic covariance function for soft measurement modeling of indoor air quality in subway stations. The results indicated that this method outperformed traditional approaches, such as partial least squares, backpropagation artificial neural networks, and least squares support vector regression, in capturing the temporal and periodic characteristics of the data [27]. However, under extreme weather conditions, air quality data may exhibit significant heteroscedasticity. To better address these non-stationary data, several studies have introduced improved GPR methods. Wang et al. introduced an enhanced hierarchical sparse Bayesian learning model that combines Gaussian kernel functions with hierarchical Bayesian models, showcasing strong generalization ability and high robustness [34]. Liu et al. proposed an improved maximum likelihood heteroscedastic Gaussian process model capable of handling non-stationary data while demonstrating excellent performance in uncertainty quantification [35]. These methods have shown remarkable performance in dealing with heteroscedastic data during extreme events, providing new insights for air quality prediction.

**Fig 1:**
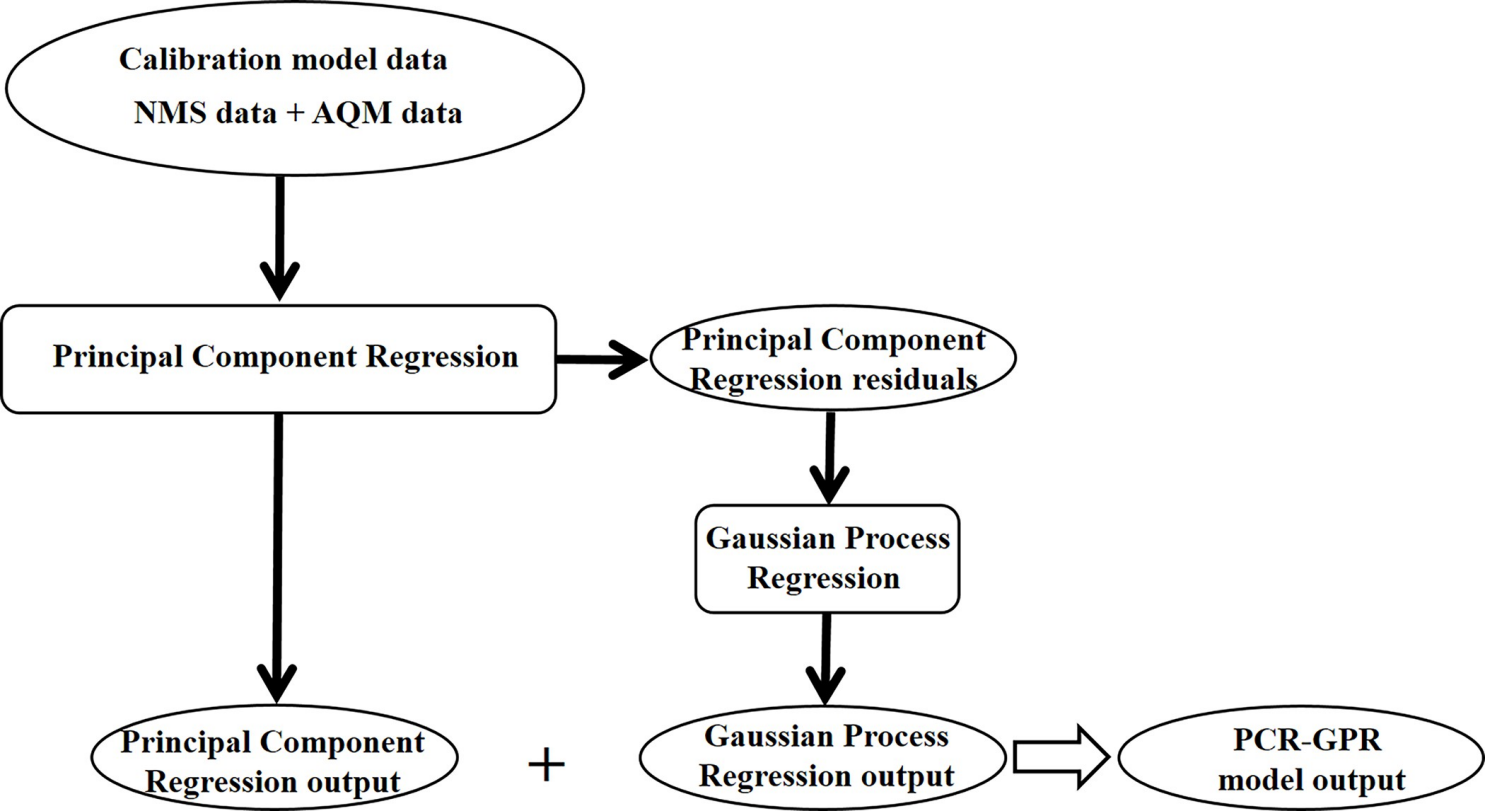
The flowchart of the regression process, where NMS data represents the pollutant concentrations measured at the NMS and AQM data represents the pollutant concentrations and meteorological parameters measured at the AQM.

Traditional statistical models offer significant advantages in terms of interpretability, clearly explaining the relationships and effects between variables. However, they have limited predictive accuracy when dealing with complex nonlinear relationships and large-scale data sets. In contrast, machine learning and neural network techniques typically provide higher predictive accuracy because of their ability to learn complex patterns and nonlinear relationships from data. However, these models are often considered to lack sufficient interpretability to understand the basis of their predictions or decisions. The aim of this study is to develop a combined model of Principal Component Regression (PCR) model and GPR, which we named PCR-GPR combined model. This model not only possesses high prediction accuracy, but also retains strong interpretability. Fig 1 depicts the construction process of the PCR-GPR combined model. This study’s results can enhance the measurement accuracy of AQMs while providing a valuable reference for air quality prediction research.

## 2. Material and methods

### 2.1. Data source and preprocessing

Major atmospheric pollutants include PM_2.5_, PM_10_, CO, NO_2_, SO_2_ and O_3_, collectively referred to as the two aerosols and four gases. Although Air Quality Monitors (AQMs) are calibrated to factory standards prior to deployment and play a crucial role in real-time and gridded monitoring of pollutant concentrations, their measurement accuracy still needs to be improved due to certain internal or external factors. Therefore, to recalibrate the AQMs, we collected two sets of measurement data from Nanjing, which originated from the China University Student Mathematical Modeling Contest (http://www.m cm.edu.cn/html_cn/node/

b0ae8510b9ec0cc0deb2266d2de19ecb.html). The first set of data came from the NMS, which recorded the concentrations of two aerosols and four gases from November 14, 2018 to June 11, 2019. The NMS measurements were stored at 1 hour intervals and contained a total of 4200 samples and were used as reference values in this study. The second set of data came from the AQM adjacent to the NMS, none of which was stored at intervals of more than five minutes and which contained a total of 234,717 samples. The AQM monitors the concentrations of the two aerosols and the four gases, while at the same time realizing the monitoring of five meteorological parameters: temperature, humidity, wind speed, pressure and precipitation.

Both sets of data required pre-processing before calibrating the AQM. Data screening revealed no missing values or outliers in the samples. Due to the short sampling interval and minute time precision of the AQM measurement data, the values of multiple measurements within the same minute show measurements at the same point in time. We averaged these duplicate values. The next step involved establishing correspondence between the data from the NMS and the AQM. To match the measurement data of the NMS, we averaged the AQM data on an hourly basis. Samples with unmatched measurement data between the NMS and the AQM were removed [36]. After pre-processing, 4144 sets of data were retained for calibrating the AQM, as displayed in Table 1.

**Table 1:** Descriptive statistics of pollutant concentrations and meteorological parameters measured by NMS and AQM after pretreatment.

Input variable	Ranges	Mean	Standard deviation	Skewness	Kurtosis	Coefficient of Variation
PM_2.5_/(μg/m^3^)	1~216.9	64.1	37.3	0.988	0.701	0.582
PM_10_/(μg/m^3^)	2~443.3	102.4	65.3	1.476	2.862	0.637
CO/(mg/m^3^)	0.05~3.895	0.863	0.452	1.463	3.136	0.524
NO_2_/(μg/m^3^)	0.947~157.1	45.2	28.4	0.653	-0.259	0.628
SO_2_/(μg/m^3^)	1~651.3	19.4	18.7	12.781	342.11	0.965
O_3_/(μg/m^3^)	0.579~259	61.6	40.9	1.091	2.035	0.665
Wind speed/(m/s)	0.133~2.387	0.7	0.346	0.862	0.748	0.494
Pressure /(Pa)	996.9~1039.8	1018.8	8.89	-0.093	-0.599	0.009
Precipitation /(mm/m^2^)	0~312.1	132.1	87	0.245	-0.728	0.659
Temperature /(°C)	-3.882~37.9	11.9	8.6	0.625	-0.399	0.724
Humidity /(rh%)	10.7~100	68.9	21.9	-0.487	-0.756	0.318

### 2.2. Data exploratory analysis

Exploratory analysis refers to the initial observation, summary, and exploration of a dataset in the process of data analysis, aiming to understand the characteristics, distribution, correlation, and other information of the data. It prepares for subsequent in-depth analysis and modeling [4, 14]. The modeling process for the two aerosols and four gases is similar since this study uses statistical modeling to achieve air quality forecasting, which is mainly based on the correlation between the data. In this paper, O_3_ is chosen as the representative of the study, and the modeling process of the remaining pollutants can be carried out in the same way.

**Fig 2:**
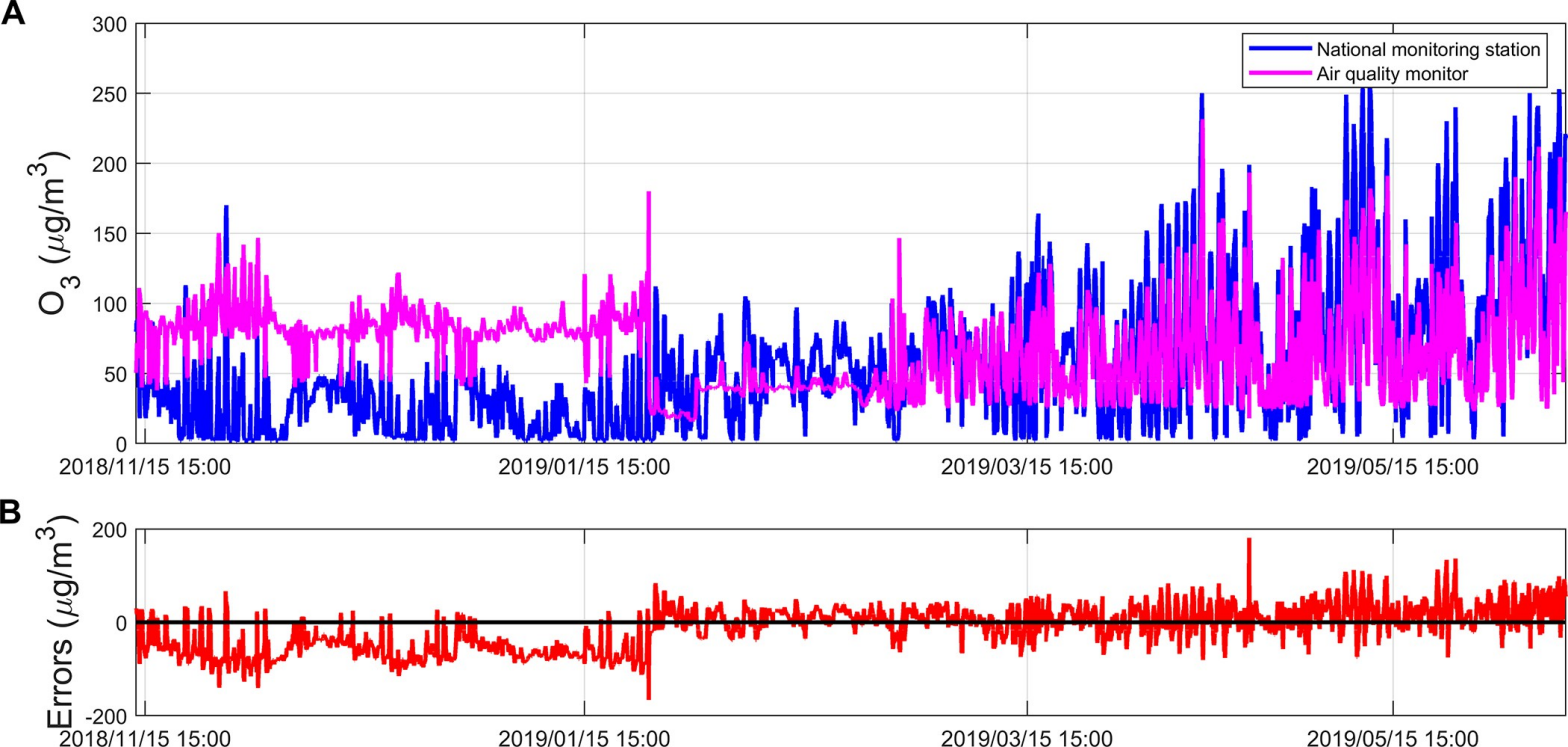
(A) Comparison of O3 concentration measurements between the NMS and the AQM; (B) Errors between the O3 concentration measurements of the NMS and the AQM. Figures are generated using Matlab (Version R2021b, https://www.mathworks.com/) [Software].

According to Fig 2, we can observe that the measurement trends of the NMS and the AQM for O_3_ concentration are generally consistent. Before January 23, 2019, the measurement results of the AQM were high relative to the NMS. However, since January 24, 2019, the measurement errors of the AQM show a positive and negative bi-directional distribution, randomly distributed around the zero point. For the distribution of measurement errors of the AQM, about 64.52% of the error values fell within the range of [–50, 50]. In addition, about 7.61% of the measurement errors had absolute values exceeding 100μg/m^3^, which indicates that the AQM is capable of performing O_3_ concentration measurements, but its measurement accuracy still needs to be improved.

**Fig 3:**
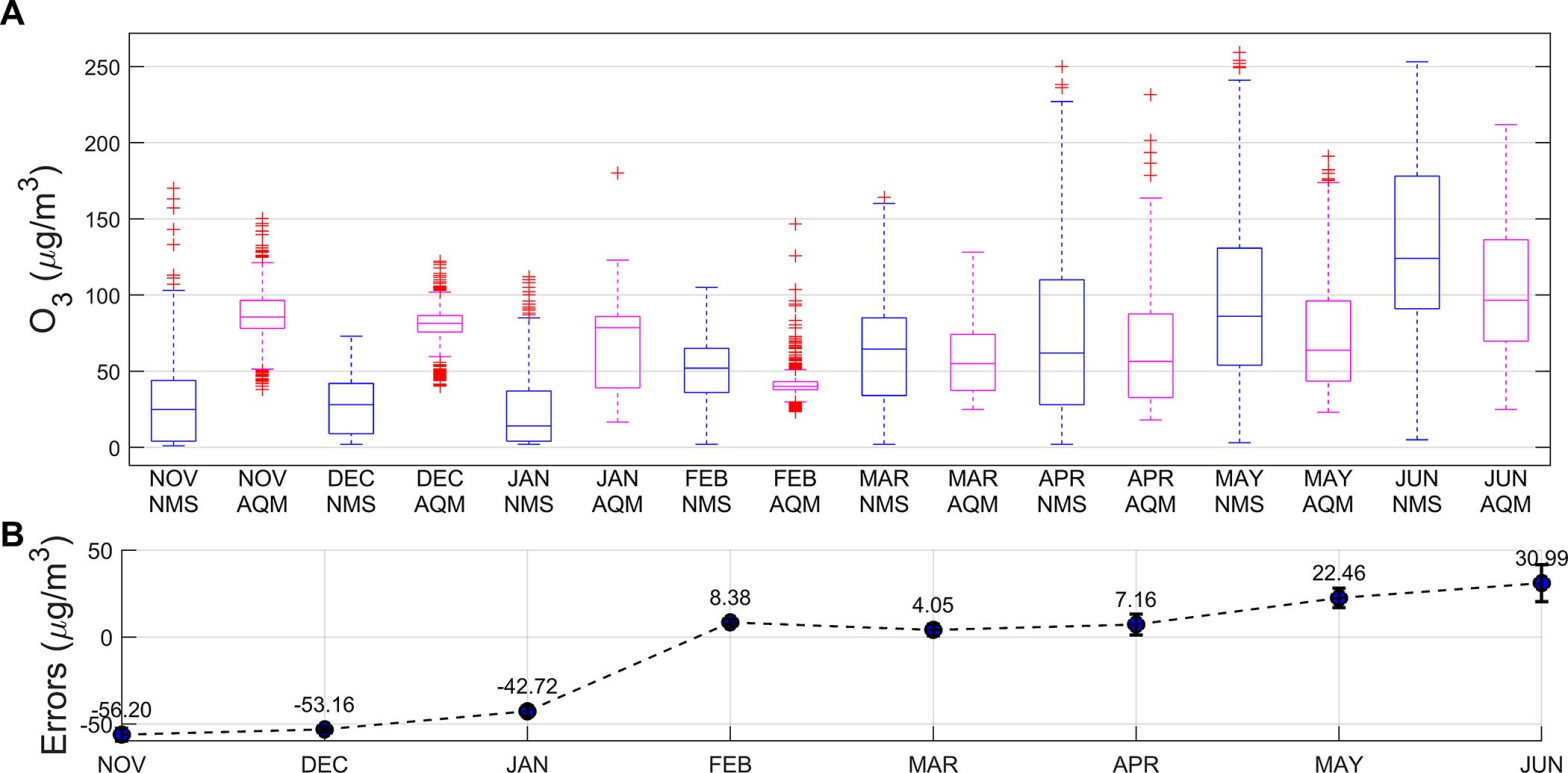
(A) Comparison of O_3_ concentration measurements between the NMS and the AQM on a monthly basis; (B) Comparison of errors for O_3_ concentration measurements between the NMS and the AQM on a monthly basis.

The measurement error of the AQM is susceptible to many external factors, such as interfering pollutants and weather conditions. In the Nanjing area, monthly variations in external factors are pronounced due to seasonal climate changes and environmental differences. To comprehend these effects, we categorized monthly measurements from both the NMS and the AQM for exploratory analysis. Fig 3 illustrates that O_3_ concentrations are lowest in January in the measurement area, attributed to lower temperatures, reduced light, and increased atmospheric stability during winter, all inhibiting O_3_ formation. The highest O_3_ concentrations occur in June, attributed to elevated temperatures, ample light, and decreased atmospheric stability during summer, which create favorable conditions for O_3_ formation and accumulation [37, 38]. In addition there are often strong photochemical reactions in the summer, such as photolysis of NO_2_ which produces O_3_. The line graph depicting the measurement error of the AQM indicates a minimum error of -56.2 μg/m^3^ in November and a maximum error of 30.99 μg/m^3^ in June. The measurement error of the AQM exhibits a rising trend over time, attributed to various internal and external factors impacting the chemical sensor during measurements.

Correlation analysis is a commonly used method to assess the degree of relationship or correlation between two or more variables [39]. Among these, the Pearson correlation coefficient is often used to measure the degree of linear correlation between two continuous variables [11]. Eq (1) is the calculation of Pearson correlation coefficient, where *x*_*i*_ and *y*_*i*_ represent the values of the variables and $\bar{x}$ and $\bar{y}$ represent the mean values of the variables. Examination of the correlation coefficients in Table 2 reveals significant correlations between pollutant concentrations measured by the NMS and those measured by the AQM, with exceptions noted for the NO_2_ concentration measured by the NMS versus the temperature measured by the AQM, and the O_3_ concentration measured by the NMS versus the CO concentration measured by the AQM. This suggests that factors influencing pollutant concentrations are highly complex. Specifically, the correlation coefficient between PM_2.5_ concentrations measured by the NMS and those measured by the AQM is 0.92, indicating a strong positive correlation. Conversely, the correlation coefficient between SO_2_ concentrations measured by the NMS and those measured by the AQM is 0.04, indicating a weak positive correlation between them.


$$r=\frac{\sum_{i=1}^{n}\left(x_{i}-\bar{x})(y_{i}-\bar{y}\right)}{\sqrt{\sum_{i=1}^{n}(x_{i}-{\bar{x})}^{2}}∙\sqrt{\sum_{i=1}^{n}(y_{i}-{\bar{y})}^{2}}}$$ (1)

**Table 2:** The Pearson linear correlation coefficients between the concentrations of the six air pollutants measured at the NMS (designated with the “R-” prefix)and the concentrations of the six air pollutants and five meteorological parameters measured at the AQM (significant correlations are indicated by * at the 0.05 level of significance).

Variable	PM_2.5_ (μg/m^3^)	PM_10_ (μg/m^3^)	CO (mg/m^3^)	NO_2_ (μg/m^3^)	SO_2_ (μg/m^3^)	O_3_ (μg/m^3^)	Wind speed (m/s)	Pres- Sure (Pa)	Preci- itation (mm/m^2^)	Temp-eratue (°C)	Humi-dity (rh%)
R-PM_2.5_/(μg/m^3^)	0.92*	0.87*	0.31*	0.32*	0.09*	0.06*	-0.24*	0.09*	-0.07*	-0.17*	0.18*
R-PM_10_/(μg/m^3^)	0.68*	0.65*	0.33*	0.31*	0.12*	0.15*	-0.18*	0.04*	-0.09*	-0.04*	-0.08*
R-CO/(mg/m^3^)	0.61*	0.58*	0.34*	0.38*	0.10*	0.10*	-0.32*	-0.08*	0.08*	-0.06*	0.22*
R-NO_2_/(μg/m^3^)	0.17*	0.08*	-0.11*	0.38*	0.10*	-0.21*	-0.35*	-0.11*	-0.14*	-0.01	-0.13*
R-SO_2_/(μg/m^3^)	0.28*	0.39*	0.51*	0.30*	0.04*	0.37*	-0.19*	0.19*	0.27*	-0.10*	0.11*
R-O_3_/(μg/m^3^)	-0.38*	-0.45*	0.03	-0.52*	-0.07*	0.42*	0.40*	-0.44*	-0.12*	0.68*	-0.61*

### 2.3. Principles of chemical sensor calibration model

PCR is a regression method based on Principal Component Analysis (PCA), which aims to reduce the dimensionality of the feature space through dimensionality reduction processing to reduce noise and redundant information in the data, thus improving the performance and generalization ability of the regression model.

PCR first requires PCA of the input features. PCA finds a new set of orthogonal bases, known as principal components, by calculating the eigenvectors and eigenvalues of the covariance matrix of the input data to represent the direction of the largest variance in the data. Then, the first k principal components are selected in PCA to reduce the original high-dimensional feature space to k-dimensional space, realizing the dimensionality reduction processing of features. This process is conducive to reducing the noise and redundant information in the data and improving the computational efficiency and generalization ability of the model. Regression models are built using regression methods such as ordinary least squares in the reduced feature space. As the feature space is reduced to the main features, the complexity of the model is reduced, while still retaining the key information in the original data, resulting in a more concise model with better explanatory performance. After building a PCR model, the model usually needs to be evaluated and tuned, and adjusted and improved as needed to obtain better predictions [40].


$$f(x)∼GP(m(x),K(x,x^{\prime }))$$ (2)

$$K(x,x^{\prime })={\left(1+\frac{{\left\|x-x^{\prime }\right\|}^{2}}{{2\alpha l}^{2}}\right)}^{-\alpha }$$ (3)

$$K(x,x^{\prime })=\sigma^{2}exp(-\frac{{\left\|x-x^{\prime }\right\|}^{2}}{{2l}^{2}})$$ (4)

$$K(x,x^{\prime })=\sigma^{2}\left(1+\frac{\sqrt{2\upsilon }}{l}|x-x^{\prime }|\right)\exp\left(-\frac{\sqrt{2\upsilon }}{l}|x-x^{\prime }|\right)$$ (5)

$$K(x,x^{\prime })=\exp\left(-\gamma {\left\|x-x^{\prime }\right\|}^{2}\right)$$ (6)

GPR algorithm is a probability-based machine learning algorithm whose basic principle is to model the data as a series of Gaussian processes and use the modeling results as the outputs, which can achieve multi-output and multi-feature prediction. The advantages of the GPR algorithm are that it can use fewer training samples, which results in higher modeling accuracy, and it has a high fitting ability, which enables multi-output prediction [41].

In the GPR model, the fitted function can be expressed as Eq (2), where *x* is the input vector referring to the concentrations of two aerosols and four gases and five meteorological parameters measured by the chemical sensor, *m*(*x*) is the given mean function, *K*(*x*, *x*′) is the covariance function at any two points *x*, *x*′ in the domain of definition. In GPR, we usually use the kernel function *K*(*r*) to represent the value of the covariance at any two points. The commonly used kernel functions are Rational Quadratic Kernel, Squared Exponential Kernel, Matérn Kernel and Exponential Kernel. Eq (3)–(6) are their expressions, where *α*, *l*, *σ*, *υ*, *γ* are hyperparameters in the kernel function.


$$p(f|x)={\left(2\pi \right)}^{\left(-N/2\right)}{\left|K\right|}^{-1/2}\exp\left(-\frac{1}{2}f^{T}K^{-1}f\right),K=K(x,x^{\prime })$$ (7)

$$p(y|f,x,\sigma^{2})=\prod_{i=1}^{N}\frac{1}{\sqrt{2\pi \sigma^{2}}}\exp\left(-\frac{{\left(y_{i}-f(x_{i})\right)}^{2}}{2\sigma^{2}}\right)$$ (8)

$$p(f|y,x)=\frac{p(y|f,x)p(f|x)}{p(y|x)}$$ (9)

The derivation of the Gaussian process is independent of which mean function is used, so we first assume *m*(*x*) = 0. The Gaussian prior distribution over the *N* target points can be written as Eq (7). Assuming that the observations *y* at point *x* are affected by the variance *σ*^2^ and are independent of each other, the likelihood function can be expressed as Eq (8), where *i* represents the *i* -th sample point. Next, we can use Bayes’ theorem (Eq (9)) to compute its posterior distribution, then Eq (10) is its log-likelihood estimate, where *I*_*N*_ denotes the unit matrix of size *N*×*N* and *N* denotes the number of observed sample points. Then the prediction of GPR f* = *f*(x*) at point x* can be expressed as Eq (11) and (12), where *E* is the approximate expected value of the function *f*(*x*), *V* is the approximate variance of the function *f*(*x*).

With the GPR model, the similarity between the training data points can be utilized to infer the distribution across the data space, which enables accurate estimation of the predicted values as well as the assessment of the prediction uncertainty.


$$logp(y|x)=\int p(y|f,x)p(f|x)du=-\frac{1}{2}y^{T}{\left(K(x,x^{\prime })+\sigma^{2}I_{N}\right)}^{-1}y-\frac{1}{2}log|K+\sigma^{2}I_{N}|-\frac{N}{2}log2\pi$$ (10)

$$E(f^{*})=K(x^{*},x){\left(K+\sigma^{2}I_{N}\right)}^{-1}y$$ (11)

$$V(f^{*})=K(x^{*},x^{*}){-K(x^{*},x)(K+\sigma^{2}I_{N})}^{-1}K(x^{*},x)$$ (12)

Taylor diagram is a graphical tool for comparing the similarity between model simulation results and observed data. It is first proposed by American meteorologist Karl E. Taylor in 2001, and it contains accuracy metrics such as correlation coefficient, standard deviation and centered root mean square difference. En (13) is the standard deviation expression where *w*_*i*_ represents the output value of the model and $\bar{w}$ is the mean value of *w*_*i*_. Eq (14) is the standard deviation and centered root mean square difference expression where *y*_*i*_ is the measured value of the NMS and $\bar{y}$ is the mean value of *y*_*i*_. Taylor diagrams are a change from the previous situation where only two metrics could be presented to represent the accuracy of a model, such as scatter plots. In a broader sense, Taylor diagrams can be extended to applications where three-dimensional data needs to be presented on a two-dimensional plane [37]. Root Mean Square Error (RMSE), Mean Absolute Error (MAE) and relative Mean Absolute Percentage Error (MAPE) are commonly used metrics to quantitatively assess the degree of closeness between the model simulation results and the observed data, and Eq (15)–(17) are their expressions.


$$\sigma =\sqrt{\frac{1}{n}\sum_{i=1}^{n}{{(w}_{i}-\bar{w})}^{2}}$$ (13)

$$E^{\prime }=\sqrt{\frac{1}{n}\sum_{i=1}^{n}{{[(y}_{i}-\bar{y}){-(w}_{i}-\bar{w})]}^{2}}$$ (14)

$$RMSE=\sqrt{\frac{1}{n}\sum_{i=1}^{n}{\left(y_{i}-w_{i}\right)}^{2}}$$ (15)

$$MAE=\frac{1}{n}\sum_{i=1}^{n}\left|y_{i}-w_{i}\right|$$ (16)

$$MAPE=\frac{1}{n}\sum_{i=1}^{n}\left|\frac{y_{i}-w_{i}}{y_{i}}\right|$$ (17)

## 3. Results

### 3.1. Results of PCR calibration model

Measurements from chemical sensors in AQMs can be effectively calibrated with the aid of air quality prediction models. However, the factors associated with the concentration of two aerosols and four gases are extremely complex. Multiple linear regression modeling was used to determine the linear relationship between the NMS and the AQM measurements. Eq (18) is the basic form of the model, where *y* is the dependent variable *x*_1_, *x*_2_,⋯,*x*_*p*_ are the independent variables, *β*_1_, *β*_2_,⋯,*β*_*p*_ are the regression coefficients of the model, and *ε* is the error term. The regression coefficients *β*_1_, *β*_2_,⋯,*β*_*p*_ indicate the effect of each independent variable on the dependent variable, and by estimating these coefficients we can assess the contribution and degree of influence of each independent variable on the dependent variable.

In this study, we divided 4144 sets of data in a ratio of approximately 4:1, with 3304 sets serving as the training set and 840 sets serving as the test set. In order to simulate the real situation and extract the seasonal variations of pollutant concentrations, we adopted a time series cross-validation method by dividing the 840 sets of test set into five consecutive time subsets, each containing 168 sets of data. Based on these subsets, we constructed five air quality forecasting models, with each model using one subset as the test set while the remaining data served as the training set. Ultimately, we combined the outputs of the test sets from these five models to generate the final testing results and averaged the outputs from the training sets of the five models to produce the final training results. This approach enables us to effectively evaluate model performance and ensure adaptability to the changing data environment. In building the first O_3_ concentration forecasting model, the O_3_ concentration measured by the NMS in the training set was used as the dependent variable, and the two aerosols and four gases concentrations measured by the AQM in the training set as well as the meteorological parameters were used as the independent variables, and the multivariate linear regression model (Eq (19)) was completed with the help of the least squares method.


$$y=Constant+\beta_{1}x_{1}+\beta_{2}x_{2}{+\cdots +\beta }_{p}x_{p}+\varepsilon$$ (18)

$$y=-1221.69+0.89x_{1}-0.509x_{2}-23.96x_{3}-0.479x_{4}+0.064x_{5}+0.455x_{6}+{20.25x}_{7}+1.22x_{8}-{0.01x}_{9}+{3.1x}_{10}-0.192x_{11}$$ (19)

The completed O_3_ concentration prediction model requires a multicollinearity diagnosis. The diagnostic results show that the maximum variance inflation factor in the model is 27.96, which exceeds 10 significantly, indicating that the model has a serious multicollinearity problem. Multicollinearity can make regression coefficient estimation unstable, reduce the accuracy of parameter estimation, and lead to overfitting. To solve this problem, PCA was used to convert the relevant independent variables into linearly independent principal components to improve model stability and generalization.

**Fig 4:**
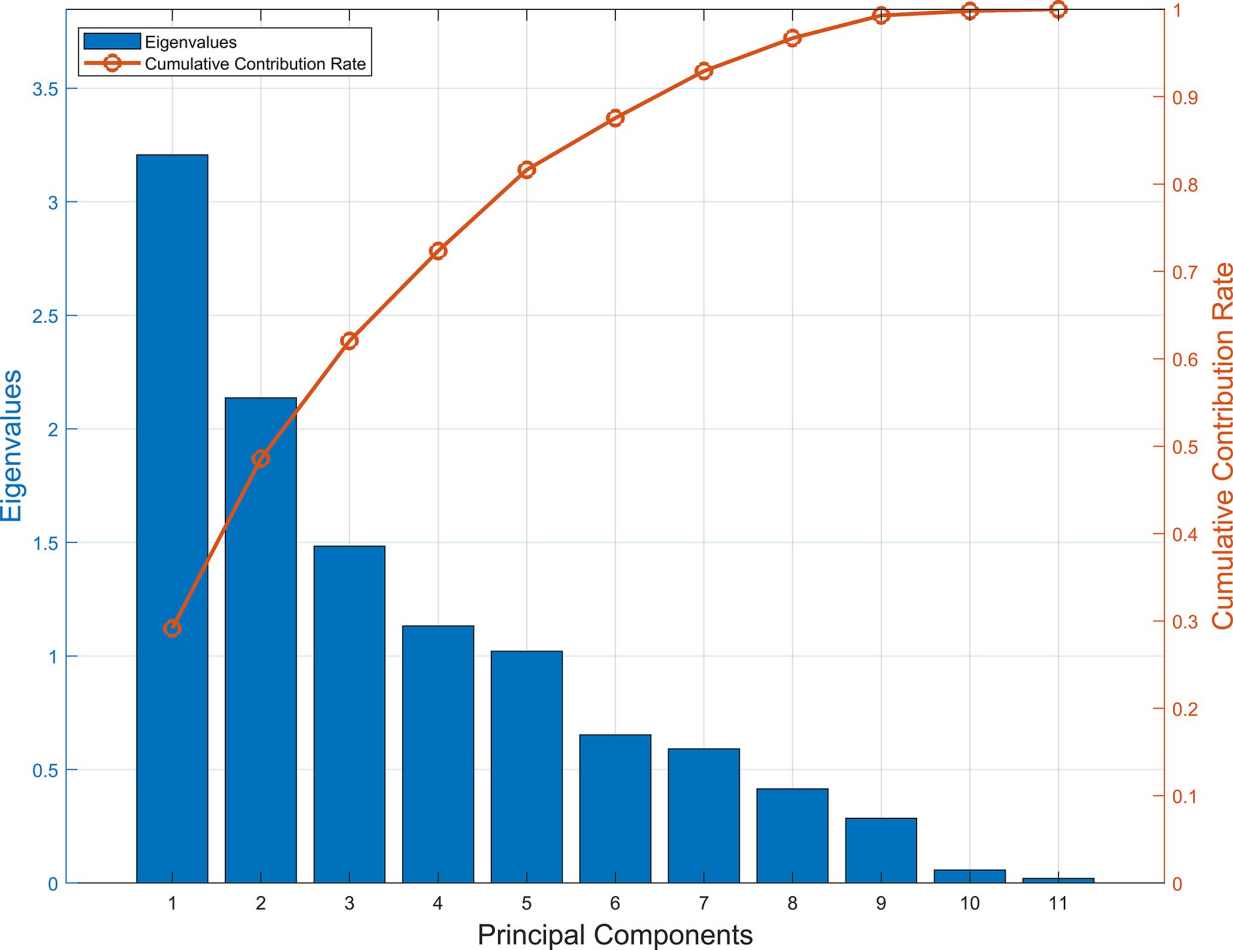
Eigenvalues and cumulative contribution rate of AQM measurements in PCA.

Fig 4 shows the details of the conversion of the AQM measurement data into principal components. It can be seen that the maximum eigenvalue of the AQM measurement data matrix is 3.21, corresponding to a contribution rate of 29.15%, and the minimum eigenvalue is 0.021, corresponding to a contribution rate of 0.19%. The cumulative contribution rate of the first 8 principal components is more than 95%, which indicates that these principal components are able to explain the degree of variability of the original data better. Using these eight principal components instead of the data measured by the AQM as the independent variables to build the O_3_ concentration prediction model can effectively solve the multicollinearity problem of the model.

With the help of SPSS 22.0, we gave the first PCR model (Eq (20)) for the prediction of O_3_ concentration. The coefficient of determination (R^2^) of the model is 0.701, indicating that 70.1% of the variation in O_3_ concentration can be explained by the model, and the model fits well overall. In the F-test of the model, the F-value is 1160.8, and the corresponding probability P-value is 0.000, which indicates that the variables introduced into the model have a significant effect on the dependent variable as a whole under the significant level α = 0.01.

Since there is a definite linear relationship between each principal component and the AQM measurements, incorporating this relationship into the PCR model allows for the variables in the PCR model to be transformed back to the original variables, thereby facilitating a better understanding and interpretation of the model results. The first O_3_ concentration prediction model and the first prediction models for the other five pollutant concentrations, after reduction to the original variables, are shown in Table 3.

**Table 3:** First PCR model of six types of air pollutant concentrations. In the model, the dependent variable is the concentration of the six pollutants at the NMS, and the independent variables are the measurements of the AQM.

Independent variable	PM_2.5_/(μg/m^3^)	PM_10_/(μg/m^3^)	CO/(mg/m^3^) (×10^−2^)	NO_2_/(μg/m^3^)	SO_2_/(μg/m^3^) (×10^−1^)	O_3_/(μg/m^3^)
Constant	292.8	83.65	889.1	145.1	-2969.8	946.2
PM_2.5_/(μg/m^3^)	0.48	0.552	0.399	0.099	0.101	0.022
PM_10_/(μg/m^3^)	0.211	0.243	0.176	0.02	0.198	-0.013
CO/(mg/m^3^)	-9.58	0.189	8.2	-32.4	361.9	-19.4
NO_2_/(μg/m^3^)	0.111	0.399	0.243	0.42	0.484	-0.56
SO_2_/(μg/m^3^)	0.043	0.138	0.121	0.098	-0.526	0.056
O_3_/(μg/m^3^)	0.003	0.073	0.045	-0.183	2	0.279
Wind speed/(m/s)	-2.05	-1.74	-16.49	-19.46	-56.93	20.77
Pressure /(Pa)	-0.27	0.033	-0.835	-0.065	2.74	-0.843
Precipitation /(mm/m^2^)	-0.04	-0.096	0.033	-0.037	0.188	0.013
Temperature /(°C)	0.276	0.511	0.607	0.167	-1.03	1.29
Humidity /(rh%)	-0.296	-0.865	-0.004	-0.486	0.715	-0.597
F value	4207.7	918.1	43600.9	408.9	3776.6	1160.8
R^2^	0.895	0.649	46.79	0.452	4.32	0.701

**Fig 5:**
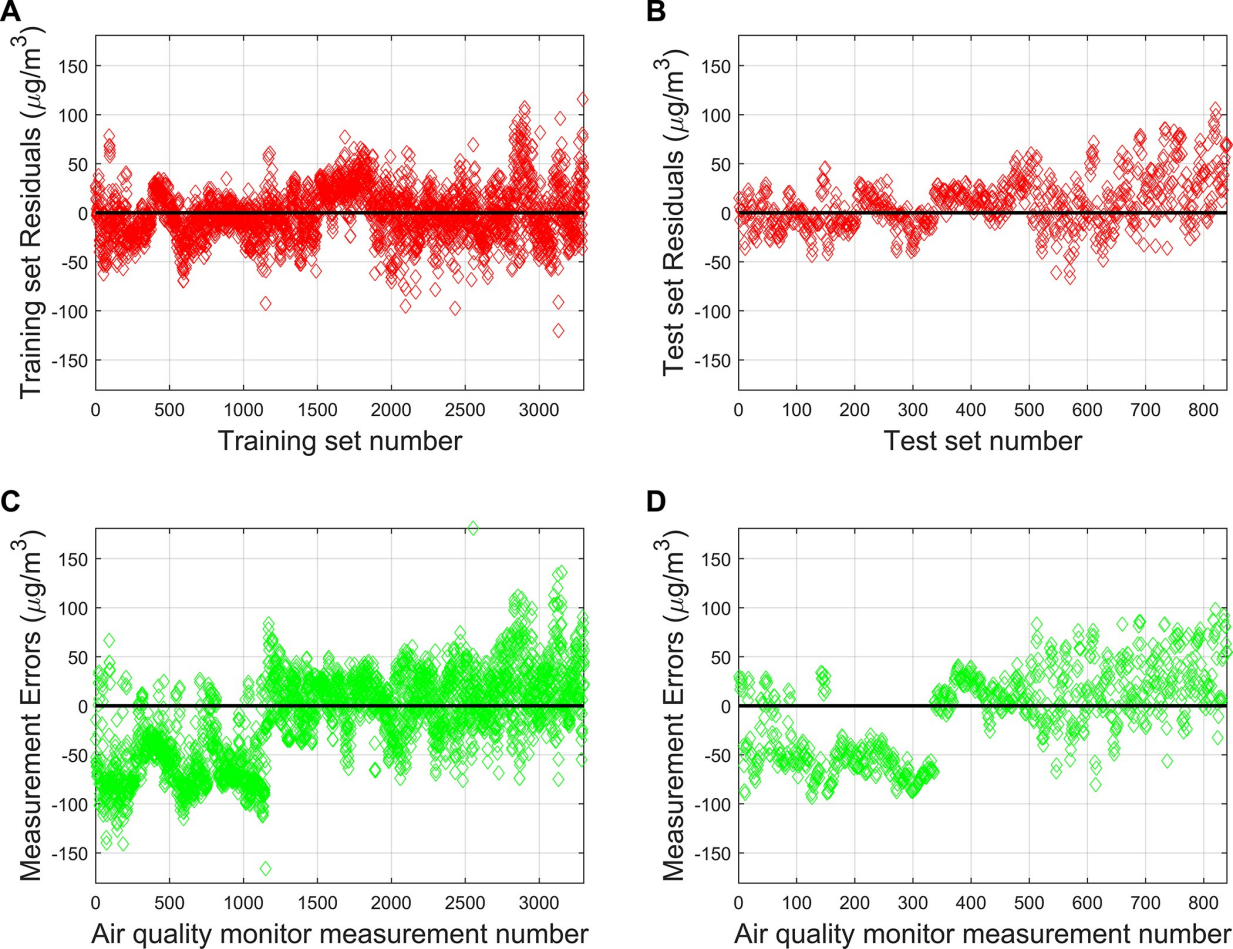
(A) Residuals of the PCR calibration model on the training set; (B) Residuals of the PCR calibration model on the test set; (C) The measurement error of the AQM at the number corresponding to the training set of the PCR calibration model; (D) The measurement error of the AQM at the number corresponding to the test set of the PCR calibration model.

Although the PCR model for O_3_ concentration prediction passed the significance test, the model fitting effect and prediction effect are also very important. To comprehensively evaluate the model’s performance, we combined the outputs of the test sets from the five PCR models to generate final prediction results and obtained the final training results by averaging the outputs of the training sets from these models. As shown in Fig 5, in the training set, the PCR model has 5 samples with absolute values of the residuals higher than 100μg/m^3^, while there are 66 samples in the corresponding AQM measurement data with absolute values of errors higher than 100μg/m^3^. There are 3,139 samples in the PCR model with absolute values of the residuals lower than 50μg/m^3^, accounting for 95.01%, while there are 2,297 samples in the corresponding AQM measurement data with absolute values of errors lower than 50μg/m^3^, accounting for 69.52%. In the test set, 1 sample in the PCR model has absolute values of residuals higher than 100μg/m^3^, while no sample in the corresponding AQM measurement data have absolute values of residuals higher than 100μg/m^3^. 775 samples in the PCR model have absolute values of residuals lower than 50μg/m^3^, accounting for 92.26% of the samples, while 549 samples in the corresponding AQM measurement data have absolute values of residuals lower than 50μg/m^3^, accounting for 65.36%. The PCR model has a certain calibration effect on the chemical sensor measurement data, and the model performs similarly in the training set and the test set, indicating that the model has a good generalization ability.


$$y=53.72-18.77p_{1}-6.01p_{2}-5.7p_{3}+12.21p_{4}+1.37p_{5}-2.56p_{6}-2.83p_{7}+11.5p_{8}$$ (20)

### 3.2. Results of PCR-GPR calibration model

PCR modeling has enabled the extraction of linear relationships between the two aerosols and four gases concentrations and their correlation factors. However, the correlations between the two aerosols and four gases concentrations and their correlates are very complex, and the nonlinear relationships between them are still hidden in the residuals of the PCR model. The GPR model as a nonparametric method is suitable for datasets of various sizes, and it is effective in capturing and modeling nonlinear relationships in the data and does not require assumptions about the distribution of the data. It is used in this study to find hidden nonlinear relationships in the residuals of PCR models.

The residuals of the first PCR model for O_3_ concentration prediction were used as the response variable, and the AQM measurements were used as the predictor variables to build the GPR model with the help of the regression learner in matlab to realize the calibration of the residuals of the PCR model. In the experiment the model validation method used default 5-fold cross-validation to combat the overfitting problem of the model.

The next step is to select the hyperparameters. In the GPR model, Basis function, Kernel function, Kernel scale, Sigma and whether to normalize the data need to be adjusted [27]. For the basis function, the software searched among Zero, Constant, and Linear. The Rational Quadratic Kernel, Squared Exponential Kernel, Matern Kernel and Exponential Kernel were the searched Kernel functions. For the search range of Kernel scale, we set it to [0.001,1]×*X*_*max*_, where *X*_*max*_ denotes the largest value in the extreme deviation of each variable in the predictor variables. The search range of Sigma was set to [0.001,10×*std*(*Y*)], where *Y* is the response variable.

Bayesian optimization is an optimization method based on Bayesian inference, which has a wide range of applications in many fields, including hyperparameter optimization, automatic machine learning, and intelligent parameter tuning. It is usually able to find a better solution in a relatively small number of iterations and thus performs well in resource-constrained or expensive optimization problems. Bayesian optimization constructs a posteriori models of the objective function by using Bayesian inference on the basis of existing observations and prior knowledge, and uses this posteriori model to guide the optimization process at each step. The core idea of this approach is to locate the places in the search space where the optimal solution is most likely to exist and to explore more in those places. It was used to implement the optimization search for the hyperparameters of the GPR model [42]. With the help of Bayesian optimization, the first GPR model hyperparameters were determined as Constant, Nonisotropic Rational Quadratic, 0.834, 0.002 and Unstandardized respectively. The residuals calibrated by the GPR model were added to the initial predictions of the PCR model to obtain the final predictions of the PCR-GPR model. In this way, we have completed the construction of the PCR-GPR model. After completing the model construction, we calculated the Spearman rank correlation coefficients between the residuals and 11 explanatory variables to validate whether the error terms of the model satisfy the assumption of homoscedasticity. At a significance level of 0.05, the results indicated that all the Spearman rank correlation coefficients were not significant (the maximum correlation coefficient was 0.02, with a corresponding p-value of 0.255). This suggests that, under the current data and model settings, there is no significant heteroscedasticity, and the constructed PCR-GPR model adheres to the basic statistical assumptions. Subsequently, the test set data can be input into the trained PCR-GPR model to predict O_3_ concentrations. Using the same method, PCR-GPR models can be obtained for all two aerosols and four gases concentrations.

**Fig 6:**
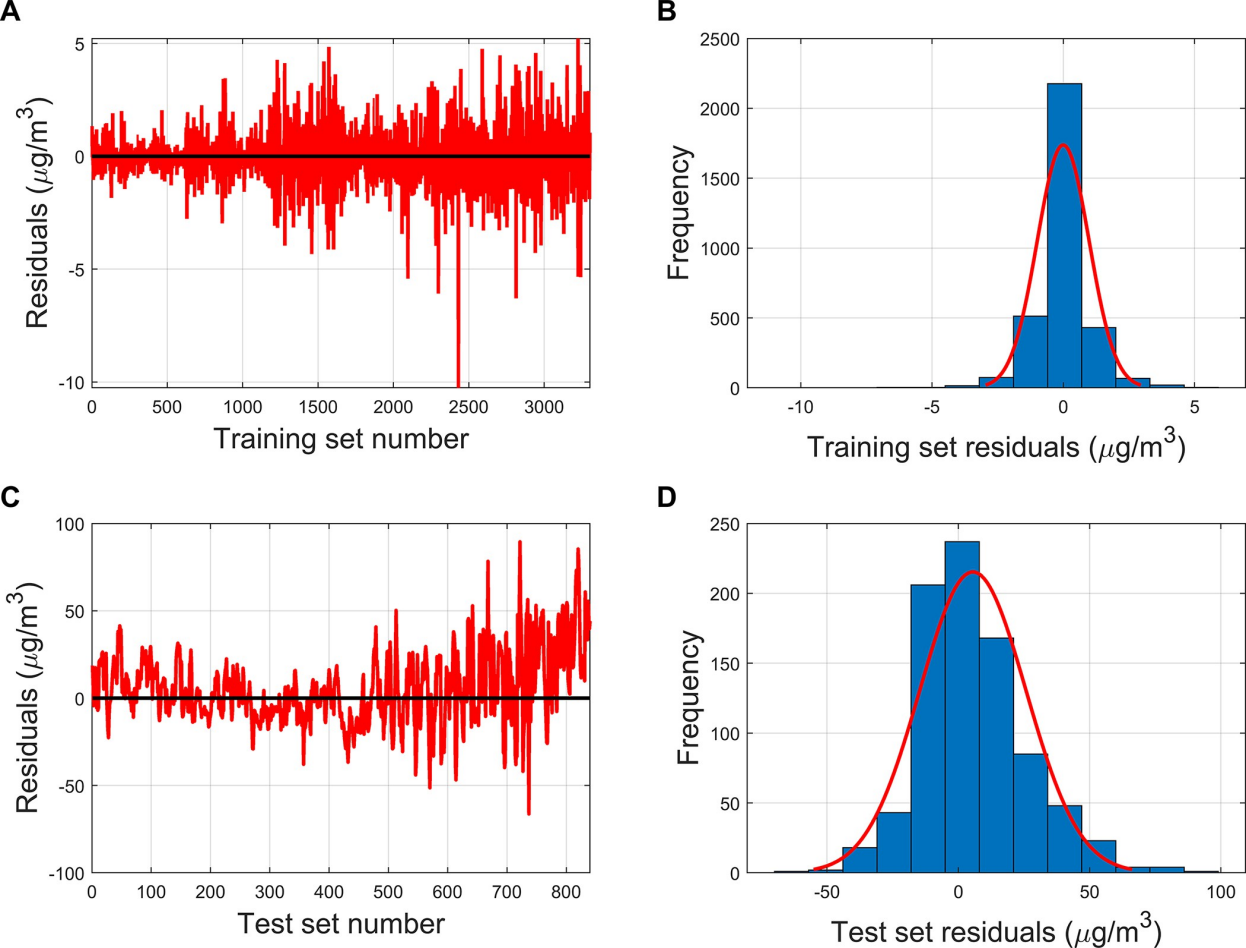
(A) The residual plot of PCR-GPR model in the training set; (B) The residual histogram of PCR-GPR model in the training set; (C) The residual plot of PCR-GPR model in the test set; (D) The residual histogram of PCR-GPR model in the test set.

Fig 6 demonstrates the residuals of the combined PCR-GPR calibrated model for O3 concentration prediction. It can be seen that the residuals of the PCR-GPR model are significantly improved compared to the PCR model, which is due to the fact that the GPR model has a better performance. In the training set, the PCR-GPR model has 3297 samples with the absolute values of the residuals lower than 5μg/m^3^, accounting for 99.78%, and 3303 samples with the absolute values of the residuals lower than 10μg/m^3^, accounting for 99.97%. In the test set, the PCR-GPR model had 371 samples with absolute values of residuals lower than 10μg/m^3^, accounting for 44.17%, and 816 samples with absolute values of residuals lower than 50μg/m^3^, accounting for 97.14%. Regardless of the training set or test set, the residuals basically obey a normal distribution and are randomly distributed around the zero point.

**Fig 7:**
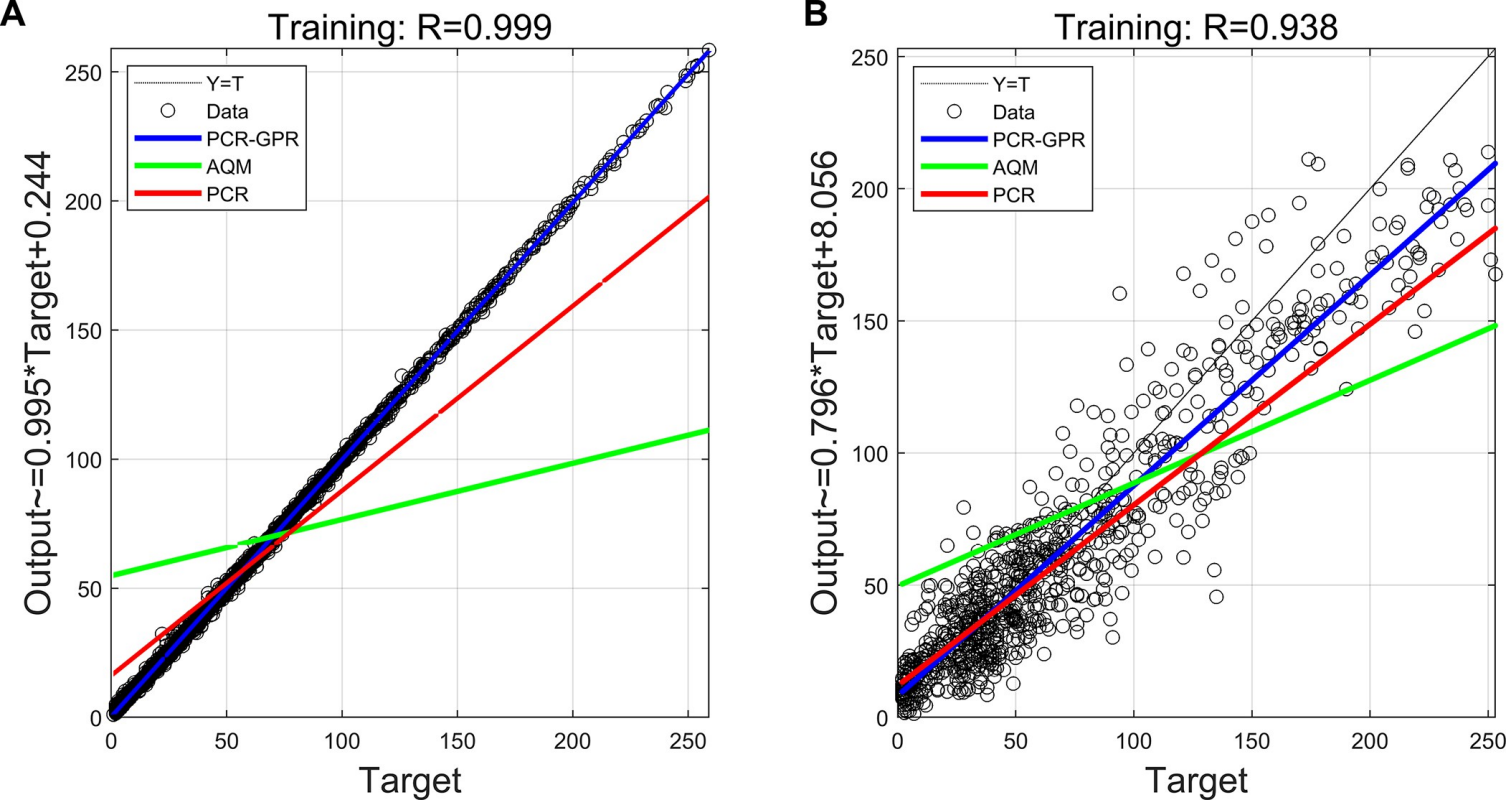
(A) The fitting effect of O_3_’s PCR-GPR model on the training set; (B) The calibration effect of O_3_’s PCR-GPR model on the test set.

Fig 7 demonstrates the regression effect of the O_3_ concentration prediction model. The linear regression line was established based on the O_3_ concentration measured at the NMS as the independent variable, and the measured data from the AQM and the model output as the dependent variable. The observation indicates that the data regression performance of the AQM is not ideal, with the PCR model showing some improvement in regressing the O_3_ concentration, while the PCR-GPR regression model demonstrates significantly better regression performance. The correlation coefficients between the target and output values in the PCR-GPR model exceed 0.93 in both the training and test sets, and the regression coefficients for both regression models are close to 1. This indicates that the output values of the PCR-GPR model are very close to the measured values of the NMS, and the model has good generalization ability.

## 4. Discussion

The PCR-GPR model enabled the calibration of the chemical sensor measurements in the AQM by predicting the O_3_ concentration. In addition, the SVR, NN and separate GPR models can also be utilized in the same way to achieve the calibration of the chemical sensor measurement data. In order to facilitate the observation of the calibration effect of each model, we showed each model in the Taylor diagram.

**Fig 8:**
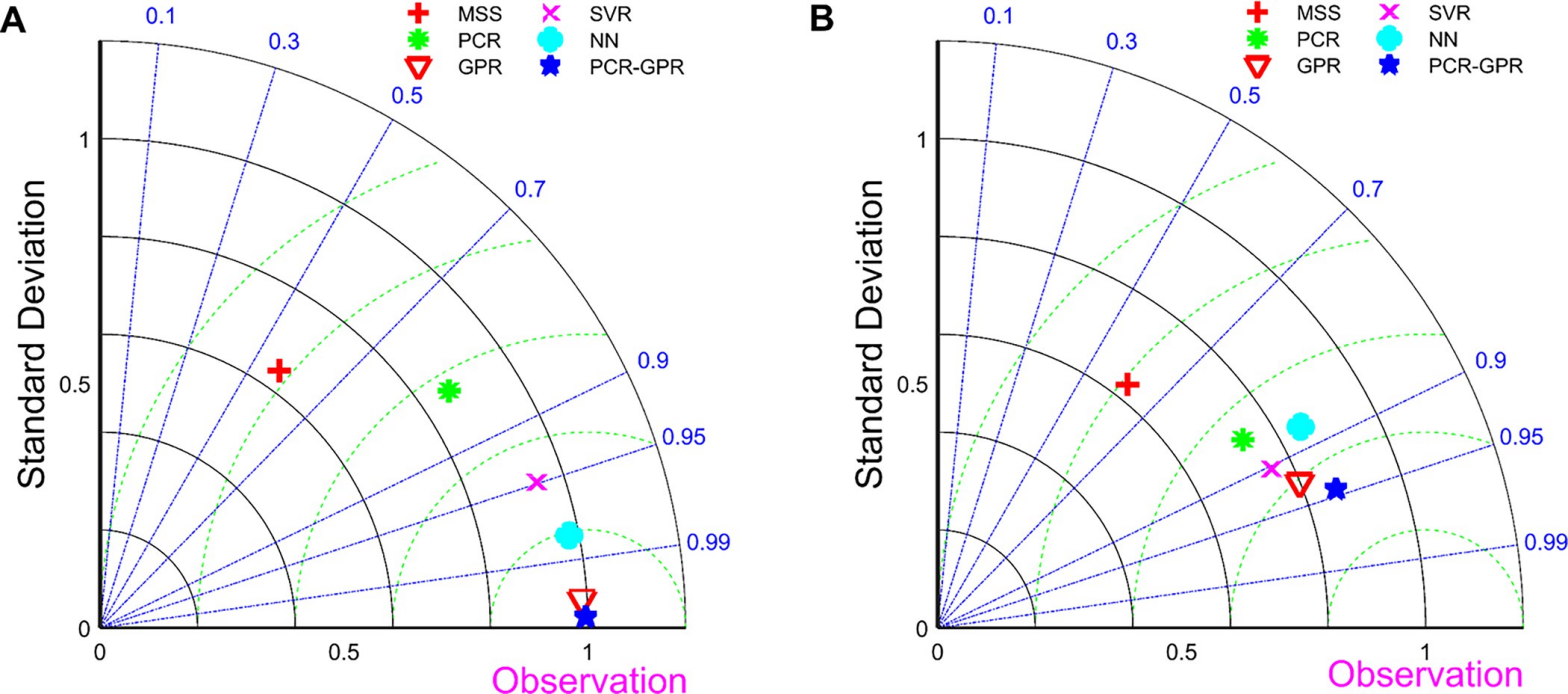
(A) Taylor diagram of the fitted O_3_ concentration values for the five calibration models on the training set; (B) Taylor diagram of the calibrated O_3_ concentration values for the five calibration models on the test set. Here AQM represents the measured values of the AQM on the corresponding set.

As can be seen in Fig 8, the AQM measurements are furthest away from the target value in both the training and test sets, indicating that the AQM measurements need to be calibrated. The PCR model has some calibration effect on the measurement data of the AQM, but still needs further improvement. SVR, NN and GPR models have better calibration effect on the measurement data of the AQM. Both in the training set and in the test set, the PCR-GPR model is closest to the target point, which indicates that the O_3_ concentration measured by the AQM is best calibrated by using the PCR-GPR model.

The Taylor diagram allows a visual comparison of the calibration effect of various models on the O_3_ concentration measured by the chemical sensor. In order to quantitatively compare the calibration effect of various models on the concentration of two aerosols and four gases measured by chemical sensors, RMSE, MAE and MAPE were introduced in this study [36]. As can be seen from the data results in Tables 4–6, the AQM exhibited the highest error values for the three measurement metrics, except for the MAPE metric for SO_2_. This highlights the need for calibration of measurement accuracy of chemical sensors. To address this issue, we compared several calibration models including SVR, PCR, NN, GPR, and PCR-GPR. The experimental results show that among these models, the PCR-GPR model exhibits the optimal performance in all evaluation metrics. The excellent performance of the PCR-GPR model is mainly attributed to the fact that it combines the advantages of the PCR model and the GPR model. The PCR model effectively extracts the linear relationship between the predictor and response variables, while the GPR model further captures the nonlinear relationship between the variables. This combination makes the PCR-GPR model both robustly interpretable and capable of handling complex nonlinear relationships, thus enabling highly accurate calibration.

**Table 4:** Comparative RMSE of AQM and various air quality calibration models on training and test sets, with NMS as reference.

Poll- utant	AQM		SVR		PCR		NN		GPR		PCR-GPR	
	Training	Test	Training	Test	Training	Test	Training	Test	Training	Test	Training	Test
PM_2.5_/(μg/m^3^)	22.45	22.99	7.93	8.6	11.4	8.92	7.85	13.68	4.38	8.66	1.69	7.89
PM_10_/(μg/m^3^)	68.18	72.64	22.33	36.71	27.5	36.78	21.14	39.25	13.92	39.09	8.49	18.9
CO/(mg/m^3^)	0.717	0.537	0.232	0.274	0.376	0.301	0.199	0.367	0.04	0.289	0.028	0.271
NO_2_/(μg/m^3^)	37.78	38.47	10.59	14.79	18.06	20.2	7.14	30.18	2.57	14.38	2.36	14.32
SO_2_/(μg/m^3^)	25.03	34.47	17.53	11.57	15.41	18.55	6	14.19	2.99	11.26	1.3	9.92
O_3_/(μg/m^3^)	47.02	45.43	14.22	26.95	25.36	27.2	8.67	29.31	2.68	23.35	0.986	20.96

**Table 5:** Comparative MAE of AQM and various air quality calibration models on training and test sets, with NMS as reference.

Poll- utant	AQM		SVR		PCR		NN		GPR		PCR-GPR	
	Training	Test	Training	Test	Training	Test	Training	Test	Training	Test	Training	Test
PM_2.5_/(μg/m^3^)	18.06	19.39	5.4	6.73	8.08	6.86	5.8	8.57	3.23	6.68	1.25	6.17
PM_10_/(μg/m^3^)	50.4	52.08	10.32	14.12	15.77	14.25	11.06	17.43	6.3	17.55	4.42	12.45
CO/(mg/m^3^)	0.583	0.43	0.159	0.227	0.283	0.258	0.148	0.291	0.027	0.235	0.019	0.223
NO_2_/(μg/m^3^)	30.31	31.28	6.77	10.77	13.77	15.9	5.06	19.27	1.65	10.59	1.55	10.36
SO_2_/(μg/m^3^)	12.75	13.72	2.61	11.2	10.48	12.74	3.96	9.16	1.68	7.94	0.744	7.09
O_3_/(μg/m^3^)	37.51	37.8	9.51	18.05	19.65	20.26	6.38	19.08	1.77	16.54	0.65	15.66

**Table 6:** Comparative MAPE of AQM and various air quality calibration models on training and test sets, with NMS as reference.

Poll- utant			SVR		PCR		NN		GPR		PCR-GPR	
	Training	Test	Training	Test	Training	Test	Training	Test	Training	Test	Training	Test
PM_2.5_/(μg/m^3^)	0.44	0.485	0.124	0.184	0.191	0.174	0.143	0.223	0.081	0.182	0.032	0.169
PM_10_/(μg/m^3^)	0.844	1.04	0.138	0.274	0.245	0.280	0.16	0.350	0.077	0.339	0.066	0.27
CO/(mg/m^3^)	0.505	0.379	0.17	0.26	0.343	0.295	0.163	0.344	0.029	0.277	0.02	0.260
NO_2_/(μg/m^3^)	2.07	2.54	0.238	0.546	0.687	0.973	0.206	1.1	0.059	0.553	0.056	0.531
SO_2_/(μg/m^3^)	0.662	0.791	0.104	0.914	0.717	1.06	0.271	0.791	0.106	0.662	0.046	0.657
O_3_/(μg/m^3^)	4.82	2.73	0.498	0.501	1.52	0.732	0.378	0.581	0.09	0.494	0.034	0.491

In terms of the specific calibration effect, although the calibration effect of the PCR-GPR model on the CO concentration measured by the chemical sensor was relatively weak in the RMSE metrics, it still reduced the metric value from 0.537 to 0.271, with an accuracy improvement of 49.53%. The calibration effect of the PCR-GPR model on the CO concentration measured by the chemical sensor was relatively weak in the MAE metric, but it also reduced the metric value from 0.43 to 0.223, with an accuracy improvement of 48.14%. In the MAPE metric, the model had a relatively weak calibration effect on the SO_2_ concentration measured by the chemical sensor, but the metric value was reduced from 0.791 to 0.657, with an accuracy improvement of 16.94%. Notably, the PCR-GPR model performed best in calibrating the PM_10_, PM_10_, and O_3_ concentrations among the three metrics for chemical sensor measurements, respectively improving accuracy by 73.98%, 76.09%, and 82.01%. In addition, the PCR-GPR model maintains a consistently high level of performance on both the training and test sets, which fully demonstrates that the model has good generalization ability. This property enables the PCR-GPR model to stably improve the measurement accuracy of chemical sensors in practical applications, which provides a strong support for the further promotion and application of air quality monitoring technology.

## 5. Conclusions

The calibration of chemical sensor measurements is important for the deployment and promotion of AQMs. In this study, the data measured by the AQM were calibrated using the PCR-GPR model with the data measured by the NMS as the baseline. The experimental results show that the PCR-GPR model performs excellently in improving the measurement accuracy of chemical sensors, and its accuracy improvement ranges from 16.94% ~ 82.01%. This model not only captures the linear relationship between the concentrations of two aerosols and four gases and the measurement data from the AQM, making it highly interpretable, but also delves deeper into the non-linear relationship between them, ensuring the model’s high accuracy. In addition, the model performs well in both the training and testing phases, which shows that it has good generalization ability. The data used in this study totaled 4,144 sets, spanning four different seasons from November 2018 to June 2019, which further verified that the PCR-GPR model could maintain high calibration accuracy in different time periods and seasons. Although the PCR-GPR model has successfully extracted linear and nonlinear relationships between the concentrations of two aerosols and four gases and the measurement data from the AQM, there are still potentially more complex relationships that have not been fully captured. Therefore, future research could consider introducing more complex mapping methods or deep learning techniques to further optimize the calibration of the model and better handle these potential relationships. At the same time, exploring integrated analysis methods for multiple sensor data or developing more adaptive algorithms to enhance the model’s adaptability and stability in dynamic environments is also a research direction worth focusing on. This will help to better understand the complex interactions of air pollutants and provide more accurate information for environmental monitoring.

## Supporting information

S1 File Air quality dataset. (XLSX)
